# Investigation of New Psychoactive Substances (NPS), Other Illicit Drugs, and Drug-Related Compounds in a Taiwanese Wastewater Sample Using High-Resolution Mass-Spectrometry-Based Targeted and Suspect Screening

**DOI:** 10.3390/molecules28135040

**Published:** 2023-06-28

**Authors:** Yuan-Chih Chen, Jen-Yi Hsu, Chih-Wei Chang, Pin-Yu Chen, Yung-Chieh Lin, I-Lin Hsu, Chiau-Jun Chu, Yen-Ping Lin, Pao-Chi Liao

**Affiliations:** 1 Department of Environmental and Occupational Health, College of Medicine, National Cheng Kung University, Tainan 704, Taiwan; 2 Public Health Bureau, Tainan City Government, Tainan 704, Taiwan; 3 Department of Food Safety/Hygiene and Risk Management, College of Medicine, National Cheng Kung University, Tainan 704, Taiwan

**Keywords:** wastewater-based epidemiology, new psychoactive substances, high-resolution mass spectrometry, suspect screening, Taiwan

## Abstract

The proliferation of new psychoactive substances (NPSs) in recent years has posed a significant challenge to public health. Traditional monitoring methods have proven insufficient in tracking these constantly evolving substances, leading to the development of alternative approaches such as wastewater-based epidemiology (WBE). The present study aims to utilize high-resolution mass spectrometry (HRMS)-based targeted and suspect screening to profile NPS, other illicit drugs, and drug-related compounds in a Taiwanese wastewater sample. For the targeted analysis, 8 out 18 standards of illicit drugs have been identified. The suspect screening approach based on approximately 3600 substances in the SWGDRUG library can further identify 92 compounds, including opiate analgesics, synthetic cathinones, phenylalkylamines derivatives, phenethylamine derivatives, tryptamine derivatives, steroids, and ephedrine-related compounds. Additionally, the presence of 5-methoxy-2-aminoindane (MEAI) in the wastewater indicates that drug dealers have recently sold this potential NPS to evade drug regulations. This study firstly reports the HRMS-based comprehensive profile of NPS, other illicit drugs, and drug-related compounds in Taiwan, which could be applied as biomarkers for estimating the consumption of drugs.

## 1. Introduction

New psychoactive substances (NPS) are defined as “substances of abuse, either in a pure form or a preparation, that are not controlled by the 1961 Single Convention on Narcotic Drugs or the 1971 Convention on Psychotropic Substances, but which may pose a public health threat” [1]. NPS are illicit or potentially illegal drugs designed to mock the effects of controlled substances. According to the 2022 World Drug Report [1], 1182 NPSs have been reported, which can be classified into synthetic cannabinoids, synthetic cathinones, ketamine and PCP-type substances, phenethylamines, piperazines, tryptamines, aminoindanes, plant-based substances, and others. The prevalence of NPSs worldwide is a significant issue and obstacle because when NPSs undergo more chemical and structural alterations, enforcing drug control laws becomes increasingly challenging [2]. Additionally, NPSs are frequently created in secret laboratories, putting addicts in danger as the content and quality of these substances are typically unknown [3]. The abuse of NPSs is also a worrying issue in Taiwan. According to the Taiwan Food and Drug Administration, more than 170 NPSs have been identified or seized in Taiwan [4]. One type of NPS, synthetic cathinones, have become the predominant NPS in Taiwan since 2017 [5]. The diversification of NPSs in both categories and items in Taiwan indicate that the early identification, surveillance, and comprehensive evaluation for NPS is necessary.

Since the quick emergence and iteration of NPSs, assessing the prevalence of NPS use through self-reported surveys or seizure data is not appropriate. Wastewater-based epidemiology (WBE) is a promising tool to estimate the dependable and timely information on geographical and temporal patterns of illicit drug use [6]. By analyzing the biomarkers of drug residues in domestic sewage, the daily mass loads of illicit drugs entering a wastewater treatment plant (WWTP) could be calculated, which can offer valuable insights into temporal trends of drug consumption, free from any self-report bias [7,8,9] or ethical concerns [10,11]. Since the application of WBE in illicit drugs analysis was first introduced in 2001 [12] and applied in 2005 [13], using WBE to monitor the use of drugs has been practiced in multiple countries and regions [14,15,16,17,18,19,20]. In addition to the documented quantitative analysis of targeted illegal drugs in sewage utilizing triple quadrupole mass spectrometry, a suspect screening strategy for illicit drug investigation using WBE and high-resolution mass spectrometry (HRMS) has been suggested [21,22,23,24]. Suspect screening can apply a database of the chemical structure of illicit drugs to screen out “suspected” signals of drugs in wastewater. These suspect features could be further subjected to MS/MS analysis to confirm their identity [25]. In the case of NPS analysis in wastewater, suspect screening can be used to monitor the broad spectrum of NPSs in wastewater with the timely information on geographical and temporal patterns, even in the absence of reference standards [26]. While some recent publications utilized suspect screening to examine NPSs in urban sewage [22,27,28,29], no similar study has been conducted in Taiwan.

For this study, we employed HRMS and suspect screening techniques to uncover the prevalence of NPSs and other illicit drugs in wastewater samples obtained from a WWTP located in southern Taiwan. The targeted analysis and suspect feature screening were conducted using the Q-Exactive Orbitrap HRMS system, while the SWGDRUG library [30], containing 3584 NPSs, other illicit drugs, and drug-related compounds, was utilized as our suspect list. In addition, we also used 18 other illicit drugs with reference standards to confirm the presence of illicit drugs in Taiwanese wastewater. To the best of our knowledge, this is the inaugural investigation utilizing suspected screening to generate a profile of NPSs and other illicit drugs in Taiwanese wastewater.

## 2. Results

### 2.1. Suspect Screening Results

The wastewater samples (240 mL) were subjected to a sample collection and preparation process, as described in Section 4.3 and Section 4.4, to filter out impurities and extract chemical compounds. The processed samples were divided into two parts: one for suspect screening and the other for targeted analysis. For the suspect screening, full-scan analysis was performed and a total of 35,472 (ESI+) and 14,991 (ESI−) features were detected in the wastewater samples. A SWGDRUG library [30] containing 3584 NPSs, other illicit drugs, and drug-related compounds was applied as the suspect list and used to match all features in the full-scan data. Features with a mass error between the suspect list and measured *m*/*z* values lower than 3 ppm were filtered as suspect features, which filtered 693 (ESI+) and 203 (ESI−) features. The suspect signals of NPSs and drugs were recorded in the inclusion list, and MS/MS analysis was triggered during data-dependent acquisition (DDA) analysis, which acquired 650 and 320 MS/MS spectra, respectively. The MS/MS spectra of suspect features were identified by matching to reference spectral databases, such as MassBank of North America (MoNA) and mzCloud. A total of 92 compounds were identified, with 64 compounds identified based on MoNA, 22 by mzCloud, and 6 by both, with matching scores over 0.7 (maximum score = 1). As the compounds involved in the SWGDRUG library were not necessarily regulated, the regulatory status of the 92 identified compounds in the United States and Taiwan was further investigated. A total of 14 substances among the 92 identified compounds were regulated in the two countries. The detailed identification results of 15 regulated substances identified in suspect screening results are listed in Table 1 and a total 92 identified compounds are shown in Table S1 (Supplementary Materials). The detailed process of suspect screening is described in Section 4.1.

### 2.2. Targeted Analysis Results

To carry out the targeted analysis, a set of 18 standards for illicit drugs, which included 15 traditional illicit drugs and 3 NPSs, were used for high-confidence identification [31]. The information on the 18 standards is listed in Table S2 (Supplementary Materials). A total of eight illicit drugs were identified, comprising six traditional illicit drugs and two NPSs. The identification results for the eight illicit drugs, including mass error, ∆RT, and MS/MS similarity scores, are presented in Table 2. The detailed process of targeted analysis is described in Section 4.1.

## 3. Discussion

### 3.1. The Identification of NPSs and Other Illicit Drugs Based on Standards

The proposed strategy successfully annotated 8 and 92 substances based on targeted and suspect screening, respectively (Table 2). The identification result of six traditional illicit drugs based on standards indicates that the prevalence of traditional drugs remains prominent in Taiwan’s drug abuse market, which corresponds to the drug seizure statistics report provided by Taiwanese government [32]. The identification of methamphetamine, amphetamine, morphine, and codeine reveals that amphetamine-type stimulants and opioids abuse in Taiwan persists to a significant extent. Ketamine was also identified in wastewater sample. The observation of ketamine signifies a prevalent trend of ketamine abuse in Taiwan [5,33].

**Figure 1:**
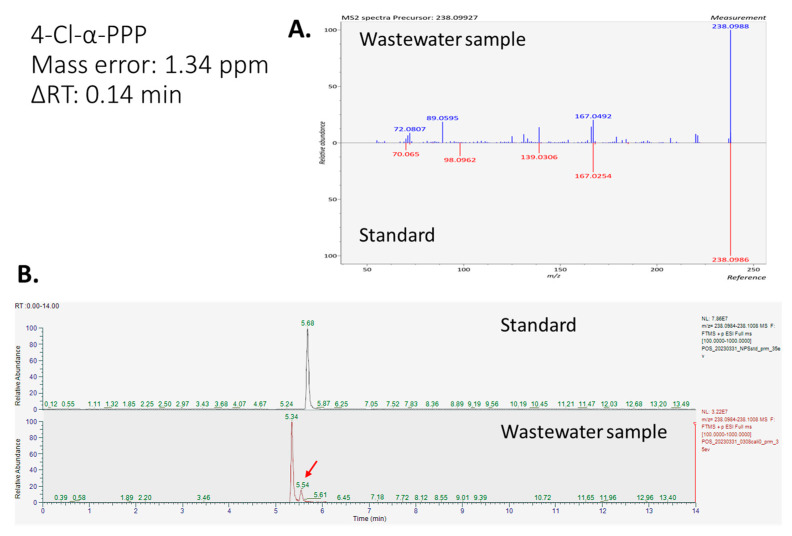
Identification result of 4-Cl-α-PPP based on standard matching. (**A**) MS/MS matching result of 4-Cl-α-PPP between standard in solvent mixture and signal in wastewater sample. (**B**) Extract ion chromatograms of 4-Cl-α-PPP in solvent mixture and wastewater sample.

The other two NPSs, mephedrone and 4-Cl-α-PPP, were also identified in wastewater samples based on matching with standards. Mephedrone is a frequently abused NPS in Taiwan, with over 13,000 cases reported by the Taiwanese government as of January 2023. The high levels of mephedrone signal in the sewage samples provide further support for its widespread use among addicts in Taiwan. Another NPS, 4-Cl-α-PPP, has also been regulated by Taiwanese government since 2019. Notably, there is no detection record of 4-Cl-α-PPP in official documents [4]. The detection of 4-Cl-α-PPP in wastewater might be possibly attributed to the recent abuse of 4-Cl-α-PPP that has not been observed by traditional methods. This circumstance highlights the potential utility of WBE in the inquiry of NPS abuse. The identification of 4-Cl-α-PPP using mass error, ∆RT, and MS/MS similarity scores is given as an example in Figure 1.

### 3.2. The Identification of Illicit Drugs Based on Suspect Screening

Based on the suspect screening analysis, a total of 92 compounds are identified. Of these, 15 substances are found to be regulated by the U.S. and Taiwanese governments, as summarized in Table 1. Opiate analgesics are the most prevalent class of compounds, comprising 6 out of the 15 regulated substances. Among them, only tramadol and dihydromorphine are reported by governmental agencies, which might indicate that the opiate analgesics might be subject to abuse recently, but remain undetected by law enforcement officials. Two synthetic cathinones, namely, 4-methyl-N,N-dimethylcathinone and MDPV, are detected in the wastewater sample. The two synthetic cathinones are also reported in the seizure report of NPSs in Taiwan (ranking 1 and 2), which indicates that the identification of NPSs from wastewater is a reliable strategy to monitor the abuse of NPS. The remaining two substances, ephedrine and N-methylephedrine, are known to be used in the synthesis and production of amphetamine and methamphetamine, which indicates that the industry of amphetamine and methamphetamine synthesis in Taiwan is still ongoing.

**Figure 2:**
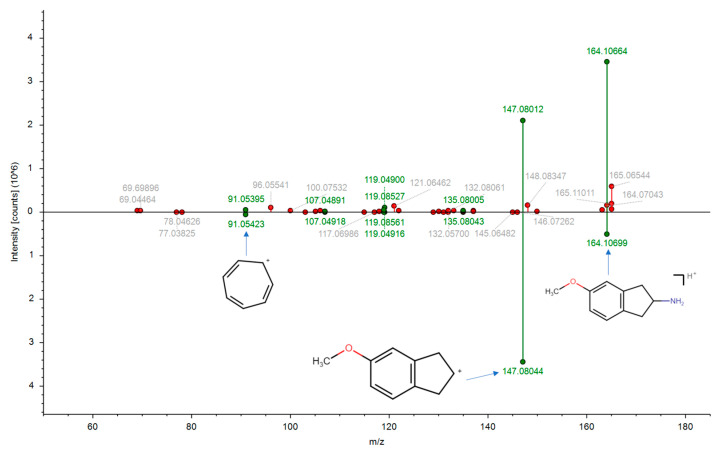
Identification result of MEAI based on MS/MS spectrum matching with mzCloud database. The MS/MS similarity score of MEAI is 0.71.

In addition to the controlled substances, a total of 78 unregulated substances are detected in the wastewater sample. Some of these substances have the potential for abuse but have not yet been subjected to regulatory control. For instance, MEAI (5-methoxy-2-aminoindane or 5-MeO-AI or Chaperon) is detected in the wastewater sample with a MS/MS similarity score of 0.71 (the MS/MS spectrum matching result is presented in Figure 2). MEAI is structurally analogous to 2-aminoindane and N-methyl-2-aminoindane, both of which are regulated in Taiwan. The identification of MEAI may be indicative of its use by drug dealers to evade Taiwan’s drug regulations.

## 4. Materials and Methods

### 4.1. Study Design

**Figure 3:**
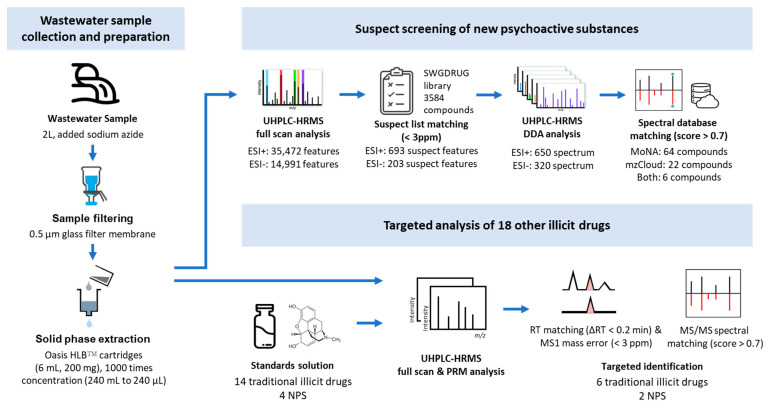
Study design of the targeted and suspect screening strategy for NPSs and other illicit drugs identification.

In this study, a targeted and suspect screening strategy was employed to identify illicit drugs and NPSs in wastewater samples. To obtain a sewage sample that is representative, we consulted the published literature [6,34,35] to design the processes for sample pretreatment and data analysis. The study design and number of filtering features at each step are presented in Figure 3.

### 4.2. Reagents and Materials

LC–MS-grade methanol (purity, ≥99.9%) was purchased from Merk (Darmstadt, Germany). HPLC-grade acetonitrile (purity, ≥99.9%) was purchased from J.T. Baker (Phillipsburg, NJ, USA). Ten standards of illicit drugs, methamphetamine (1 mg/mL), amphetamine (1 mg/mL), ketamine (1 mg/mL), norketamine (1 mg/mL), morphine (0.1 mg/mL), codeine (1 mg/mL), Tetrahydrocannabinol (THC, 1 mg/mL), 11-nor-9-carboxy-THC (THC-COOH, 0.1 mg/mL), 3,4-methylenedioxymethamphetamine (MDMA, 1 mg/mL), mephedrone (1 mg/mL), flunitrazepam (1 mg/mL), 7-aminoflunitrazepam (1 mg/mL), nimetazepam (1 mg/mL), 7-aminonimetazepam (1 mg/mL), and zopiclone (1 mg/mL) were purchased from Cerilliant (Round Rock, TX, USA). Three standards of NPSs, 4′-chloro-alpha-pyrrolidinopropiophenone (4-Cl-α-PPP, 1 mg, purity, ≥98%), 4′-Fluoro-α-pyrrolidinopentiophenone (4-F-α-PVP, 1 mg, purity, ≥98%), and 1-pentyl-3-(2-bromophenylacetyl)indole (JWH-249, 1 mg, purity, ≥98%) were purchased from Cayman Chemicals (Ann Arbor, MI, USA). Sodium azide (500 mg, purity, ≥99%) was purchased from Sigma-Aldrich (St. Louis, MO, USA).

### 4.3. Sample Collection

Three barrels of wastewater samples (500 mL each) were collected in a WWTP in southern Taiwan at 3:00 p.m., 7 March 2023. The WWTP is capable of processing 75,000 m^3^ of wastewater per day, providing service to approximately 513,000 people in the city. Twenty liters of wastewater samples were collected at influent of the WWTP after the filter of solid impurities. After transferring and mixing three samples into one 2 L aliquot of a polyethylene terephthalate bottle, anti-bacterial agents, approximately 100 mg of sodium azide, were added. The mixed sample was then stored at a temperature of 4 °C before extraction and analysis.

### 4.4. Sample Preparation

The collected wastewater sample was first filtered to remove solid impurities using a 0.5 μm glass filter membrane. The resulting filtered sample (240 mL) was then divided into four equal parts of 60 mL each, and each part was subjected to solid phase extraction using Oasis HLB™ cartridges (6 mL, 200 mg) from Waters^®^ (Milford, MA, USA). To prepare the cartridges for extraction, 6 mL of methanol was added to each cartridge for conditioning, followed by the addition of 3 mL of deionized water for equilibration. After equilibration, 60 mL of the filtered sewage sample was loaded onto each cartridge and washed with 4 mL of a 5% methanol/deionized water solution. Finally, the extracted sample was eluted using 4 mL of methanol, and the resulting eluate was collected in four glass tubes and dried. After drying the extracted wastewater sample with automatic nitrogen evaporator, all four glass tubes were reconstituted with a total of 240 μL 5% acetonitrile/deionized water solution, and then centrifuged at 15,000× *g* for 15 min at 4 °C. The resulting supernatant was transferred to a sample vial and stored at −20 °C until it could be analyzed using ultra high-performance liquid chromatography–high-resolution mass spectrometry (UHPLC–HRMS).

### 4.5. UHPLC–HRMS Analysis

UHPLC–HRMS analyses were performed using a Dionex UltiMate 3000 UPLC system connected to an Orbitrap Q Exactive Plus Hybrid mass spectrometer (Thermo Fisher Scientific, Bremen, Germany). An amount of 5 μL from each sample was loaded onto a Phenomenex Luna Omega polar C18 column (100 × 2.1 mm, 1.6 µm, Torrance, CA, USA) and maintained at 40 °C. A 5 μL sample was loaded into LC–HRMS in each analysis. The UHPLC was operated at a flow rate of 250 μL/min and maintained at 45 °C with the solvent system (solvent A: deionized water containing 0.1% formic acid; solvent B: 100% acetonitrile containing 0.1% formic acid) and eluent gradient (2% B for 0–1 min; 2–99% B in 1–11 min; 99% B in 11–13 min; 99–2% B in 13.01–14 min, and then resumed to 2% B for 1 min for pressure equilibration).

For the HRMS analysis, data were acquired in both positive and negative mode. The full-scan data with 100 to 1000 *m*/*z* scanning range was performed at 70,000 FWHM with automatic gain control (AGC) = 3 × 10^6^ and the max injection time was 200 ms. Tandem mass spectrometry analysis was performed in PRM mode for targeted analysis and DDA mode with an inclusion list for suspect screening. For the PRM mode, resolution was 70,000, normalized collision energy of higher-energy collisional dissociation (HCD) was 35%; AGC = 1 × 10^5^, the isolation window was 1.6 *m/z*, and the max injection time was 200 ms. For the DDA analysis, resolution was 17,500, normalized collision energy of higher-energy collisional dissociation (HCD) was 35%, AGC = 1 × 10^5^, the isolation window was 1.6 *m/z*, intensity threshold was 1.6 × 10^5^, and the max injection time was 50 ms. Mass-to-charge ratio value of 18 illicit drug standards and suspect features matching to the SWGDRUG library in MS1 data were listed in the inclusion list of PRM and DDA analysis, respectively.

### 4.6. Data Processing and Drugs Identification

For the targeted analysis, PRM analysis results of wastewater samples were processed with MS-DIAL [36] software for peak picking and peak identification. The raw data of the wastewater sample and solvent with 18 standards mixture were firstly transferred into “.abf” files and inputted into MS-DIAL. The criteria of peak height were set at 10,000 amplitude and S/N at 10. The aligned peak table of wastewater samples and standards mixture with both MS1 and MSMS information was generated by MS-DIAL and outputted. If the features in the wastewater sample shared similar retention time (∆RT < 0.2 min), *m*/*z* (Δ*m*/*z* < 3 ppm), and MS/MS spectrum (similarity score calculated by MS-DIAL > 0.7, with a maximum score of 1), these compounds were considered as level 1 confidence identification [31]. For the suspect screening, raw UHPLC–HRMS full-scan data of wastewater samples were also processed with MS-DIAL for peak picking. The criteria were identical to the values performed in the targeted analysis. A suspect list of 3584 NPSs, other illicit drugs, and drug-related compounds was constructed based on the SWGDRUG library [30] and matched with MS1 features in the full-scan data of wastewater samples. The complete suspect list contains 3584 drugs and drug-related compounds, presented in Table S3 (Supplementary Materials). When the features in wastewater samples matched to the molecular weight of 3584 compounds with mass error lower than 3 ppm, these features were filtered and listed in the inclusion list of DDA for acquiring MS/MS spectra. The DDA data of wastewater sample were identified by reference spectrum matching with MoNA spectrum database (https://mona.fiehnlab.ucdavis.edu, accessed on 1 March 2023) and mzCloud drug of abuse/illegal drugs category (https://www.mzcloud.org/DataViewer, accessed on 1 March 2023). The similarity scores between experimental MS/MS spectra and reference spectra in databases were calculated based on the MS/MS similarity calculation algorithm built in MS-DIAL and Compound Discoverer software, respectively. The identification results with the similarity score higher than 0.7 were considered as level 2~3 confidence identification [31].

## 5. Conclusions

The identification of NPSs, other illicit drugs, and drug-related compounds in wastewater is critical for discovering biomarkers of drug consumption patterns within a community. This research successfully detected 8 and 92 substances through targeted and suspect screening methods in wastewater treatment plants (WWTPs) located in southern Taiwan, including 23 regulated substances. Among these substances, the identification of MDAI, not previously recorded through traditional survey methods, indicates the potential value of analyzing wastewater using comprehensive target and suspect screening methodologies for illicit drug research and control. This is the first investigation in Taiwan to utilize suspect screening to create a profile of illicit drugs in wastewater. The evaluation of the temporal and spatial distribution of drug abuse in Taiwanese sewage can be fully assessed in the future with the completion of more comprehensive sewage sample sampling and the identification of additional illicit drugs.

## Figures and Tables

**Table 1:** Identification of illicit substances (n = 15) regulated in U.S. and Taiwan in wastewater samples.

Class	Compound Names	Molecular Formula	Measured *m/z*	Mass Error	MS/MS Similarity Scores	Classification in U.S.	Classification in Taiwan
Opiate analgesics	Tapentadol	C_14_H_23_NO	222.1847	−2.4	0.76	2	2
	Meperidine	C_15_H_21_NO_2_	248.1642	−0.2	0.82	2	2
	Levorphanol	C_17_H_23_NO	258.1848	−1.7	0.86	2	2
	Tramadol	C_16_H_25_NO_2_	264.1956	−0.8	0.86	4	4
	Hydromorphone	C_17_H_19_NO_3_	286.1433	−1.6	0.83	2	2
	Dihydromorphine	C_17_H_21_NO_3_	288.1590	−1.5	0.78	1	2
Synthetic cathinones	4-Methyl-N,N-dimethylcathinone	C_12_H_17_NO	192.1381	−1.0	0.75	-	3
	Methylenedioxypyrovalerone, (MDPV)	C_16_H_21_NO_3_	276.1591	−1.2	0.74	1	2
Phenethylamine derivatives	5-(2-Aminoethyl)-2,3-dihydrobenzofuran (5-AEDB)	C_10_H_13_NO	164.1068	−1.2	0.70	-	3
Tryptamine derivatives	Bufotenin	C_12_H_16_N_2_O	205.1335	−0.2	0.70	1	3
Phenylalkylamines derivatives	N-methyl-2-aminoindane	C_10_H_13_N	148.1121	−0.5	0.78	-	3
Steroids	1,4-Androstadiene-3,17-dione	C_19_H_24_O_2_	285.1845	−1.4	0.87	3	-
	Methenolone	C_20_H_30_O_2_	303.2314	−1.5	0.74	3	-
Others	Ephedrine	C_10_H_15_NO	166.1225	−0.8	0.93	-	4
	N-Methylephedrine	C_11_H_17_NO	180.1382	0.5	0.90	-	4

**Table 2:** Identification of traditional illicit drugs and NPS (n = 8) in wastewater sample based on standards.

Class	Compound Name	Molecular Formula	Theoretical *m/z* ([M + H]^+^)	Measured *m/z* ([M − H]^+^)	Mass Error (ppm)	∆RT (min)	MS/MS Similarity Scores
Traditional drugs	Amphetamine	C_9_H_13_N	136.1121	136.1123	1.52	0.03	0.78
	Methamphetamine	C_10_H_15_N	150.1277	150.1278	0.50	0.01	0.95
	Norketamine	C_12_H_14_ClNO	224.0837	224.0838	0.59	0.07	0.76
	Ketamine	C_13_H_16_ClNO	238.0993	238.0992	−0.42	0.05	0.99
	Morphine	C_17_H_19_NO_3_	286.1438	286.1435	−1.00	0.52	0.84
	Codeine	C_18_H_21_NO_3_	300.1594	300.1598	1.27	0.06	0.74
NPS	Mephedrone	C_11_H_15_NO	178.1226	178.1227	0.55	0.09	0.73
	4′-chloro-alpha-pyrrolidinopropiophenone (4-Cl-α-PPP)	C_13_H_16_ClNO	238.0993	238.0996	1.34	0.14	0.78

## Data Availability

The data presented in this study are available in the article and supplementary material.

## Supplementary Materials

The following supporting information can be downloaded at: https://www.mdpi.com/article/10.3390/molecules28135040/s1, Table S1: 92 identified substances based on suspect screening; Table S2: Information of 18 standards for high confidence identification; Table S3: the complete suspect list contains 3584 drugs and drug-related compounds.
