# Supplementary material for: Investigation of New Psychoactive Substances (NPS), Other Illicit Drugs, and Drug-Related Compounds in a Taiwanese Wastewater Sample Using High-Resolution Mass-Spectrometry-Based Targeted and Suspect Screening
Source: Molecules. 2023 Jun 28;28(13):5040. doi: 10.3390/molecules28135040 (PMC10343774; doi:10.3390/molecules28135040)
Supplement: Supplementary file 1 [file molecules-28-05040-s001.zip › Supporting Information_Table S1_S2_20230626-comments.pdf]

Supporting Materials

Investigation of New Psychoactive Substances (NPS), Other Illicit Drugs, and Drug-Related Compounds in a Taiwanese Wastewater Sample Using High-Resolution Mass-Spectrometry-Based Targeted and Suspect Screening

Yuan-Chih Chen1, Jen-Yi Hsu1, Chih-Wei Chang1, Pin-Yu Chen1, Yung-Chieh Lin1, I-Lin Hsu2, Chiau-Jun Chu2, Yen-Ping Lin2 and Pao-Chi Liao1,3,*

1 Department of Environmental and Occupational Health, College of Medicine, National Cheng Kung University, Tainan 704, Taiwan

2 Public Health Bureau, Tainan City Government, Tainan 704, Taiwan

3 Department of Food Safety/Hygiene and Risk Management, College of Medicine, National Cheng Kung University, Tainan 704, Taiwan

[*]Correspondence:

Dr. Pao-Chi Liao

Department of Environmental and Occupational Health

National Cheng Kung University College of Medicine

138 Sheng-Li Road, Tainan 70428, Taiwan

TEL: 886-6-2353535 ext 5566, FAX: 886-6-2743748

E-mail: liaopc@mail.ncku.edu.tw

Table S1. 92 identified substances based on suspect screening.

Compound name	Molecular formula	Theoretical m/z ([M+H]$^+$)	Measured m/z ([M-H]$^+$)	Mass error (ppm)	MS/MS similarity scores	Database
Paracetamol	$C_8H_9NO_2$	152.0706	152.0703	-2.0	0.97	mzCloud
Ephedrine	$C_{10}H_{15}NO$	166.1226	166.1225	-0.8	0.93	mzCloud
11-ketotestosterone	$C_{19}H_{26}O_3$	303.1955	303.1949	-1.9	0.88	mzCloud
Dextrorphan	$C_{17}H_{23}NO$	258.1852	258.1849	-1.3	0.88	mzCloud
1,4-Androstadiene-3,17-dione	$C_{19}H_{24}O_2$	285.1849	285.1845	-1.4	0.87	mzCloud
Tramadol	$C_{16}H_{25}NO_2$	264.1958	264.1956	-0.8	0.86	mzCloud
Levorphanol	$C_{17}H_{23}NO$	258.1852	258.1848	-1.7	0.86	mzCloud
Testosterone propionate	$C_{22}H_{32}O_3$	345.2424	345.2420	-1.2	0.85	mzCloud
Hydromorphone	$C_{17}H_{19}NO_3$	286.1438	286.1433	-1.6	0.83	mzCloud
Phenethylamine	$C_8H_{11}N$	122.0964	122.0965	0.6	0.82	mzCloud
Anthranilic acid	$C_7H_7NO_2$	138.0550	138.0549	-0.4	0.82	mzCloud
N-Methyl-2-phenylpropan-1-amine	$C_{10}H_{15}N$	150.1277	150.1278	0.5	0.82	mzCloud
5alpha-Androstan-3,6,17-trione	$C_{19}H_{26}O_3$	303.1955	303.1949	-1.9	0.81	mzCloud
6beta-Hydromorphol	$C_{17}H_{21}NO_3$	288.1594	288.1590	-1.5	0.81	mzCloud
1,3-Dimethylbutylamine	$C_6H_{15}N$	102.1277	102.1277	-0.3	0.80	mzCloud
Diphenhydramine	$C_{17}H_{21}NO$	256.1696	256.1692	-1.5	0.80	mzCloud
N-Methyl-2-Al	$C_{10}H_{13}N$	148.1121	148.1120	-0.5	0.78	mzCloud
Formestane	$C_{19}H_{26}O_3$	303.1955	303.1949	-1.9	0.78	mzCloud
Dihydromorphine	$C_{17}H_{21}NO_3$	288.1594	288.1590	-1.5	0.78	mzCloud
Tapentadol	$C_{14}H_{23}NO$	222.1852	222.1847	-2.4	0.76	mzCloud
4-Methyl-N,N-dimethylcathinone	$C_{12}H_{17}NO$	192.1383	192.1381	-1.0	0.75	mzCloud
Testosterone cypionate	$C_{27}H_{40}O_3$	413.3050	413.3042	-2.0	0.74	mzCloud
Methenolone	$C_{20}H_{30}O_2$	303.2319	303.2314	-1.5	0.74	mzCloud
MDPV	$C_{16}H_{21}NO_3$	276.1594	276.1591	-1.2	0.74	mzCloud
(-)-Caryophyllene oxide	$C_{15}H_{24}O$	221.1900	221.1899	-0.4	0.73	mzCloud
MEAI	$C_{10}H_{13}NO$	164.1070	164.1068	-1.2	0.72	mzCloud
Bufotenin	$C_{12}H_{16}N_2O$	205.1335	205.1335	-0.2	0.70	mzCloud
5-AEDB	$C_{10}H_{13}NO$	164.1070	164.1068	-1.2	0.70	mzCloud
Nicotine	$C_{10}H_{14}N_2$	163.1230	163.1227	-0.7	0.97	MoNA
Cyclohexylamine	$C_6H_{13}N$	100.1121	100.1120	0.1	0.96	MoNA
Cimetidine	$C_{10}H_{16}N_6S$	253.1230	253.1224	-1.3	0.95	MoNA
Phenylacetaldehyde	C_8H_8O	121.0648	121.0649	1.8	0.95	MoNA
4-Methyl-5-thiazoleethanol	C_6H_9NOS	144.0478	144.0475	-0.9	0.94	MoNA
Carbamazepine	$C_{15}H_{12}N_2O$	237.1023	237.1020	0.0	0.94	MoNA
Atenolol	$C_{14}H_{22}N_2O_3$	267.1703	267.1700	-0.2	0.94	MoNA
Trimethoprim	$C_{14}H_{18}N_4O_3$	291.1452	291.1446	-0.9	0.94	MoNA
Sulfapyridine	$C_{11}H_{11}N_3O_2S$	250.0645	250.0643	0.3	0.94	MoNA
Progesterone	$C_{21}H_{30}O_2$	315.2319	315.2314	-0.4	0.94	MoNA
Amantadine	$C_{10}H_{17}N$	152.1434	152.1432	-0.2	0.93	MoNA
Salicylamide	$C_7H_7NO_2$	138.0550	138.0548	-0.2	0.93	MoNA
Bisoprolol	$C_{18}H_{31}NO_4$	326.2326	326.2320	-0.8	0.93	MoNA
Caffeine	$C_8H_{10}N_4O_2$	195.0877	195.0873	-0.8	0.93	MoNA
4-Androstene-3,17-dione	$C_{19}H_{26}O_2$	287.2006	287.2002	-0.2	0.93	MoNA
Methocarbamol	$C_{11}H_{15}NO_5$	242.1023	242.1021	0.2	0.92	MoNA
Levetiracetam	$C_8H_{14}N_2O_2$	171.1128	171.1125	-0.8	0.92	MoNA
Pilocarpine	$C_{11}H_{16}N_2O_2$	209.1285	209.1284	0.7	0.92	MoNA

Theobromine	$C_7H_8N_4O_2$	181.0720	181.0717	-0.7	0.91	MoNA
THEOPHYLLINE	$C_7H_8N_4O_2$	181.0720	181.0717	-0.7	0.91	MoNA
Ethambutol	$C_{10}H_{24}N_2O_2$	205.1911	205.1909	0.2	0.91	MoNA
Dextromethorphan	$C_{18}H_{25}NO$	272.2009	272.2005	-0.4	0.91	MoNA
Lidocaine	$C_{14}H_{22}N_2O$	235.1805	235.1802	-0.2	0.91	MoNA
N-Methylephedrine	$C_{11}H_{17}NO$	180.1383	180.1382	0.5	0.90	MoNA
Celecoxib	$C_{17}H_{14}F_3N_3O_2S$	382.0832	382.0826	-0.4	0.90	MoNA
Piperine	$C_{17}H_{19}NO_3$	286.1438	286.1433	-0.6	0.90	MoNA
Androsta-1,4-Dien-3,17-Dione	$C_{19}H_{24}O_2$	285.1849	285.1844	-0.7	0.90	MoNA
Phenylalanine	$C_9H_{11}NO_2$	166.0863	166.0861	0.0	0.90	MoNA
Adenine	$C_5H_5N_5$	136.0618	136.0618	1.1	0.90	MoNA
Flecainide	$C_{17}H_{20}F_6N_2O_3$	415.1451	415.1442	-1.1	0.89	MoNA
Fexofenadine	$C_{32}H_{39}NO_4$	502.2952	502.2941	-1.1	0.88	MoNA
Propranolol	$C_{16}H_{21}NO_2$	260.1645	260.1642	-0.2	0.88	MoNA
Diethyl-phthalate	$C_{12}H_{14}O_4$	223.0965	223.0961	-0.7	0.88	MoNA
Acetaminophen	$C_8H_9NO_2$	152.0706	152.0705	0.3	0.88	MoNA
Citalopram	$C_{20}H_{21}FN_2O$	325.1711	325.1707	-0.1	0.87	MoNA
Hexylamine	$C_6H_{15}N$	102.1277	102.1277	0.6	0.85	MoNA
Metronidazole	$C_6H_9N_3O_3$	172.0717	172.0716	0.6	0.85	MoNA
Hordenine	$C_{10}H_{15}NO$	166.1227	166.1226	0.7	0.85	MoNA
3`-Aminoacetophenone	C_8H_9NO	136.0757	136.0757	1.0	0.84	MoNA
Fluconazole	$C_{13}H_{12}F_2N_6O$	307.1114	307.1111	0.2	0.84	MoNA
Cyclophosphamide	$C_7H_{15}Cl_2N_2O_2P$	261.0321	261.0320	0.7	0.83	MoNA
Phenylethanolamine	$C_8H_{11}NO$	138.0914	138.0913	0.6	0.82	MoNA
N-Methyl-2-pyrrolidone	C_5H_9NO	100.0757	100.0756	0.0	0.82	MoNA
Meperidine	$C_{15}H_{21}NO_2$	248.1645	248.1642	-0.2	0.82	MoNA
Harmol	$C_{12}H_{10}N_2O$	199.0866	199.0865	0.5	0.81	MoNA
Tryptamine	$C_{10}H_{12}N_2$	161.1073	161.1075	2.1	0.81	MoNA
Nalidixic acid	$C_{12}H_{12}N_2O_3$	233.0921	233.0919	0.3	0.81	MoNA
Terbutaline	$C_{12}H_{19}NO_3$	226.1438	226.1434	-0.6	0.79	MoNA
Venlafaxine	$C_{17}H_{27}NO_2$	278.2115	278.2112	0.1	0.79	MoNA
Alpha-pyrrolidinovalerophenone	$C_{15}H_{21}NO$	232.1696	232.1692	-0.7	0.78	MoNA
(R)-(-)-Phenylephrine	$C_9H_{13}NO_2$	168.1019	168.1019	0.9	0.76	MoNA
Indole	C_8H_7N	118.0651	118.0651	0.7	0.76	MoNA
Canrenone	$C_{22}H_{28}O_3$	341.2111	341.2106	-0.5	0.74	MoNA
Thymol	$C_{10}H_{14}O$	151.1118	151.1117	0.7	0.73	MoNA
Norgestrel	$C_{21}H_{28}O_2$	313.2162	313.2157	-0.6	0.73	MoNA
Linoleic acid	$C_{18}H_{32}O_2$	281.2475	281.2472	-0.1	0.72	MoNA
Phenelzine	$C_8H_{12}N_2$	137.1073	137.1073	0.8	0.72	MoNA
Betaxolol	$C_{18}H_{29}NO_3$	308.2220	308.2216	-0.3	0.71	MoNA
Phenacetin	$C_{10}H_{13}NO_2$	180.1019	180.1018	0.4	0.71	MoNA
Thymidine	$C_{10}H_{14}N_2O_5$	243.0976	243.0973	0.0	0.70	MoNA
Benzoic acid	$C_7H_6O_2$	121.0281	121.0284	-2.5	0.90	MoNA
4-Chlorophenol	C_6H_5ClO	126.9943	126.9945	-1.7	0.90	MoNA
Salicylic acid	$C_7H_6O_3$	137.0232	137.0233	-0.9	0.80	MoNA
Acetominophen	$C_8H_9NO_2$	150.0546	150.0550	-2.4	0.92	MoNA

Table S2. Information of 18 standards for high confidence identification.

Compound name	Molecular formula	Theoretical m/z ([M+H]$^+$)	Type of drugs
Methamphetamine	$C_{10}H_{15}N$	150.1277	Traditional drugs
Amphetamine	$C_9H_{13}N$	136.1121	Traditional drugs
Ketamine	$C_{13}H_{16}ClNO$	238.0993	Traditional drugs
Norketamine	$C_{12}H_{14}ClNO$	224.0837	Traditional drugs
Morphine	$C_{17}H_{19}NO_3$	286.1438	Traditional drugs
Codeine	$C_{18}H_{21}NO_3$	300.1594	Traditional drugs
THC[1]	$C_{21}H_{30}O_2$	315.2319	Traditional drugs
THC-COOH[1]	$C_{21}H_{28}O_4$	345.2061	Traditional drugs
MDMA[1]	$C_{11}H_{15}NO_2$	194.1176	Traditional drugs
Mephedrone	$C_{11}H_{15}NO$	178.1227	Traditional drugs
Flunitrazepam	$C_{16}H_{12}FN_3O_3$	314.0936	Traditional drugs
7-Aminoflunitrazepam	$C_{16}H_{14}FN_3O$	284.1194	Traditional drugs
Nimetazepam	$C_{16}H_{13}N_3O_3$	296.1030	Traditional drugs
7-Aminonimetazepam	$C_{16}H_{15}N_3O$	266.1288	Traditional drugs
Zopiclone	$C_{17}H_{17}ClN_6O_3$	389.1124	Traditional drugs
4-Cl-α-PPP[1]	$C_{13}H_{16}ClNO$	238.0993	NPS
4-F-α-PVP[1]	$C_{15}H_{20}FNO$	250.1602	NPS
JWH-249[1]	$C_{21}H_{22}BrNO$	384.0958	NPS

1. Names are abbreviated, full names are in section 5.1
